# Thermal Protective Properties and Breathability of Multilayer Protective Woven Fabrics for Wildland Firefighting

**DOI:** 10.3390/polym14142967

**Published:** 2022-07-21

**Authors:** Ana Kalazić, Snježana Brnada, Ana Kiš

**Affiliations:** 1 Department of Textile Design and Management, Faculty of Textile Technology, University of Zagreb, Prilaz baruna Filipovića 28a, 10000 Zagreb, Croatia; akalazic@ttf.unizg.hr; 2 Vertiv Croatia d.o.o., 10000 Zagreb, Croatia; akis@ttf.unizg.hr

**Keywords:** protective textile, woven fabric, porosity, breathability, thermal protection, wildland firefighters, aramid fibers, comfort properties

## Abstract

A firefighter in the wildland fields spends an average of 8 to 16 h during which he encounters enormous physical effort and very demanding outdoor conditions of high temperatures. Research shows that the most common injuries are due to the occurrence of heat stress, and not due to lack of protection against burns. Therefore, for this very specific field of firefighting, it is necessary to provide clothing that will, in addition to adequate flame protection, provide good comfort properties such as lightweight suits, good porosity and breathability, so that gaseous sweat and heat generated by body heating can be released into the environment. The aim of this study was to determine the influence of structural parameters of multi-weft woven fabrics on two mutually contradictory properties—breathability and thermal protection. When designing fabrics, the goal was to produce a structure with a high proportion of volume pores, which, regardless of the increased volume of the fabric, insure the fabric mass would be acceptably small. Volume pores in the fabric have two roles—as a heat insulator and as an inhibitor of the breathability of the material. The analysis of the obtained results showed that the thickness and mass of the fabric have a greater influence on the water vapor resistance, while the heat transmission property is more affected by the thickness, porosity and fiber content.

## 1. Introduction

Requirements for firefighting clothing and equipment depend on the specifics of the firefighting specialty: outdoor firefighting, firefighting in buildings and cars, firefighting in petrochemicals, gas stations, etc., [1].

The requirements for firefighting clothing are numerous. A firefighter in the field can spend 8 to 16 h for dealing with outdoor fires. Therefore, in addition to protection against high temperatures and flames, there are requirements for wearing comfort (increased mobility, breathability). The two most important factors are maximizing thermal fire protection and minimizing metabolic heat stress. Thermal protection is of primary importance for firefighting protective clothing, but at the same time, its comfort and the thermal stress that the firefighter feels during the active extinguishing should be taken into account [2]. These two requirements are opposed to each other, because fabrics with higher thermal protection, consequently, have low breathability and high stiffness.

Nowadays, the most commonly used fibers for making thermal protective clothes are meta-aramid and para-aramid fibers. Both types of fibers have very high thermal stability, while para-aramid fibers are additionally characterized by high mechanical properties, so they are often used for ballistic purposes [3].

Composite materials for fire protection in today’s market consist of several layers. The first outer layer is a fabric of aluminized aramid (standard weaves-plain, twill and satin). Beneath the outer fabric is a thick layer of non-woven carbon-fiber textile. The third layer is a vapor-permeable membrane; however, the question arises as to how much of its functionality is below the thick first and second layers, and the last layer of lining of the FR treated fabric. Such composites provide high thermal protection, however, the problem is low thermophysiological comfort, which is especially evident in interventions in extreme conditions, such as extinguishing fires in open spaces. Mass, stiffness and bulkiness of clothing gives an additional burden to a subject that can increase the production of metabolic heat in stressful conditions, which are typically associated with firefighting and emergency reactions [4]. Such protective clothing has greater insulation due to the thermal protection it must provide, which in turn leads to the production of metabolic heat. The reason for this is that the protective function of heat can only be achieved by sacrificing a certain degree of comfort, and a balance between protection and comfort is necessary for each type of protective clothing [2].

The relationship between the structural and constructional characteristics of fabrics is the subject of scientific research. Some authors have proposed modeling tools for predicting the protective thermal properties of multi-layer and single-layer materials depending on their structural and construction parameters [5,6].

In this work, the influence of fabric thickness, mass, porosity, and fiber content of muti-weft fabrics on water vapor resistance and heat transfer properties will be investigated. Therefore, much of the development in this research is driven by a desire to improve the comfort properties of the material without sacrificing user protection (e.g., lighter, more vapor-permeable, more flexible, etc.).

## 2. Wildland Fire Fighting

Extinguishing wildland fires is an extremely demanding occupation that takes place mainly during the summer season [7]. Activities performed during firefighting require work with hand tools of various weights (3–20 kg). Those actions are usually performed in difficult conditions, such as inhaling combustion by-products, working on steep terrain and in hot environments. All these circumstances together contribute to the high physiological stress observed during forest-fire fighting. Performing demanding tasks in hot environments is associated with increased heat stress. The additional use of personal protective clothing can increase the heat load of wildland firefighters and, consequently, limit their performance.

Personal protective clothing has a negative effect on thermoregulatory mechanisms for heat dissipation, as it limits heat loss and vapor transfer between the skin and the environment. Nevertheless, personal protective clothing protects firefighters from a variety of work-related hazards, mainly due to heat exposure. They are made according to safety standards (ISO, 2003), where the technical requirements for fabrics are specified.

The degree of thermal and vapor permeability insulation of protective clothing is dependent on the thickness of the clothing, the trapped layers of air and the characteristics of the fibers. The performance of thermal protective clothing is affected by many variables, including environmental conditions (temperature, humidity, wind speed, etc.), the nature of the textile used (fabric structure, weight and thickness, fiber type, etc.), heat transfer mechanisms (convection, conductivity, thermal radiation), and the presence of moisture [8,9].

Moisture inside the clothing system can come from internal or external sources. For wildland fires, indoor moisture usually includes sweat produced by the firefighter. Additionally, outdoor moisture consists of rain or dew, water spray from a hose, and/or swamp or lake water that a firefighter must walk through. The effect of moisture on heat transfer through the exhaust system may depend on the degree of moisture absorption, the location of moisture in the system, the place on the body, its source (internal or external), the time of moisture application (before, during or after exposure to heat) and the duration of received heat.

Normal working hours for wildland firefighting are 8–16 h a day, where they are exposed to an average heat flux of 1–8 kW/m^2^ [10]. In extreme fire conditions, they can be exposed to air-heat fluxes in the range of 20 to 100 kW/m^2^. When extinguishing fires in places where synthetic building materials are located, the range of air-heat flows to which the firefighter may be exposed can be even higher. While extinguishing fires, flash fires represent a very high potential danger that firefighters may face. The heat intensity of such fires is about 80 kW/m^2^ and lasts for a few seconds. Such high heat flux can lead to heat injuries or life-threatening injuries.

Fire-fighting protective clothing for wildland firefighting must meet the following requirements (according to ISO TC 94/SC 14/WG 3). It should provide protection from thermal radiation, reduce the risk of injury from burns, and reduce the possibility of heat exhaustion. Protective clothes should be light and comfortable, porous and breathable (permeable to gaseous sweat approximately 1–2 L of sweat/h). Additionally, it is important to dissipate metabolic heat, and maintain thermal balance and comfort in a wide range of fire intensities, climatic conditions and duration of work.

For protection and comfort purposes, firefighting protective clothing used for structural fire extinguishing usually consists of several layers [11]. It consists of a flame-resistant outer layer and a thermal membrane consisting of a moisture barrier, a thermal barrier, and a cladding material. The outer layer is an aluminized surface that reflects the heat of radiation and provides thermal resistance, mechanical resistance to cuts, tearing and abrasion, and flame resistance [12]. Inherently non-combustible materials, flame-retardant fibers such as para- and meta-aramids, polybenzimidazole (PBI) and some blends are used as the outer layer. PBI fiber (developed by Celanese) can absorb more moisture than cotton and has a comfort-rating equal to that of 100% cotton materials. Some fibers are flame retardant (FR) treated (products such as Proban^®^ and Pyrovatex^®^) to improve performance.

Firefighters in wildland fires generally wear single-layer clothing that usually consists of only an outer layer that protects against heat radiation and direct flame. Single layer clothing provides comfort by ensuring optimal temperature in the microclimate between the material layer and the body of the firefighter working long hours in dry, warm weather conditions.

### Heat Transfer

There are two types of heat transfer: dry heat transfer and evaporative heat transfer.

Given the great variety of outdoor environments where fires can occur, clothes of firefighters who deal with wildland fires must be versatile to prepare them for any environment they may encounter. Its primary function must be protection against thermal radiation [13].

The protective clothing that firefighters must wear when extinguishing wildland fires could be heavy. Although its primary role is to protect them from external thermal influences, it also retains the heat released by the evaporation of sweat (in the area between the firefighter’s body and the layers of protective clothing). The environment of the area affected by fire often has a high level of humidity, which causes a decrease in the rate of evaporation [14]. The greater the weight and thickness of the garment, the greater the negative impact of moisture on a person, and by increasing the humidity in the environment, the firefighter will endure a shorter time in the field before experiencing symptoms of heat stress [15]. The amount of time a firefighter can spend in such conditions is affected by his condition and the acclimatization of the clothing (breathability, ability to change air/steam). The breathability of protective clothing is reduced by increasing the mass and layers that prevent the flow of air and water vapor through the material. In addition to clothing, firefighters who put out wildland fires must wear additional equipment that increases the weight and further prevents the movement of air inside the garment and the evaporation of sweat from the skin. For example, backpacks retain air between their numerous straps etc.

The body transfers heat minimally through conduction. *Conduction* is the contact heat transfer created by touching surrounding objects. Because firefighters in fire-affected open areas rarely touch objects in the environment, this type of heat transfer is rarely pronounced. Unlike conduction, a large amount of heat transfer occurs by convection, i.e., the transfer of heat by the movement of hot air along the surface of the subject’s skin. As air passes through the surface of the skin, it either dissipates some of the heat if the air is colder or adds heat if the air is warmer than the skin. *Radiation* is the transfer of heat due to temperature difference between the skin and the surfaces of surrounding objects. Heat transfer is of particular importance in extinguishing fires in open spaces in conditions where firefighters work in the sun or near fire, whereby heat is transferred to them from these facilities. All these types of heat transfer to clothing are collectively called *dry heat transfer*. The standard unit of measurement for dry heat transfer is W/m^2^ and it is used to determine the value of clothing insulation in m^2^ K/W, defined as the resistance to dry heat transfer (Rct). 

The total value of thermal insulation includes all layers located between the skin and the environment, including separate layers of textile materials and layers of air between them. Layers of air in the pores or air pockets of textile material are very important for thermal insulation because static air is a very good insulator of heat and makes up more than half of the usual insulation of clothing. When adding more layers of clothing, the air inside these layers should be taken into account as this increases the thermal insulation. Clothing insulation is often expressed in units of Clo, which is equal to 0.1555 m^2^ K/W. It is equivalent to the amount of insulation that would “balance the heat produced by a person at rest in normal indoor climates” wearing a business suit with the heat loss due to evaporation (sweating) and breathing.

*Evaporative heat transfer* corresponds to the maximum amount of sweat that can be produced and sustained by the average human body, which is approximately 1 L/h. In the sweat evaporation compensable zone, the body must use sweat evaporation to cool down the body. If the body cannot meet the amount of evaporation required to cool down (E_req_) due to water content in the air or impermeability of the clothing being worn, then the heat in the body will continue to build up. Breathability is usually described in terms of evaporative resistance (Ret) expressed in units of kPa m^2^/W. The permeability index (i_m_) is another way of describing the amount of evaporation that can occur through a garment. It ranges from 0 (totally impermeable) to 1 (totally permeable), and is often expressed as the Permeability index ratio (i_m_/clo).

Firefighters cope well with extreme conditions in which survival is a higher priority than comfort. However, comfort also affects their survival; therefore, it is an important aspect of protective firefighting clothing. During a typical firefighting day, a firefighter walks on uneven terrain (climbs and descends), builds defensive lines, removes flammable dry plants (branches, shrubs, etc.), works with a chainsaw and sets backfires [16].

The Standard on Protective Clothing and Equipment for Wildland Fire Fighting and Urban Interface Fire Fighting (NFPA 1977) is the official standard for regulating the design and level of protection of protective clothing in outdoor firefighting. It was created in response to the requirements for a special standard made especially for outdoor firefighting, as firefighters in wildfire environments face completely different challenges than others. The sufferings and injuries of firefighters in open spaces are most commonly due to the occurrence of heat stress, and not due to lack of protection against burns. For this reason, when designing clothing for firefighters of this category, it is necessary to provide thermal protection from external heat sources with fire-resistant clothing and equipment, without causing an excessive load of internal heat stress [17]. The standard specifies a limit of a total of 18 maximum permissible layers of material on the clothing of firefighters who extinguish fires in open areas to avoid heat stress. This refers to layers of material that are added to the garment, such as pockets, knee and elbow pads, and trimmings, which can affect the amount of heat that can pass through the garment.

## 3. Porosity of Woven Fabrics

**Figure 1:**
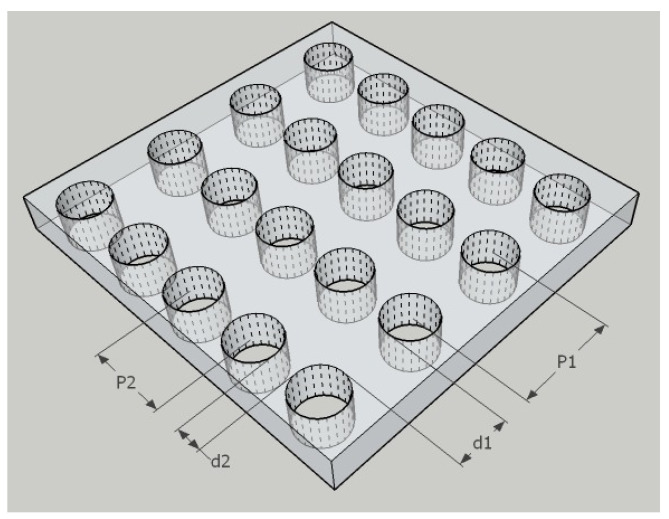
Ideal model of porous woven fabric structure (d = thread thickness, *p* = distance between threads, 1 and 2 denote warp or weft) Adapted from [18].

When the woven fabric is observed as a three-dimensional formation, empty spaces (pores) can be found in the fibers, between the fibers in the yarns and between the warp and weft threads in the fabric [18]. As textile materials, fabrics have, unlike knitwear or nonwovens, the most precisely defined internal geometric model of porous structure. It is in the form of a tubular system, with each macro pore having a cylindrical shape with a permanent cross-section over its entire length. Figure 1 shows the ideal model of the porous structure of the woven fabric.

The following parameters are usually used to compare fabrics with different porosity: pore cross-sectional area, pore areal distribution, pore density, equivalent pore diameter, maximum and minimum pore diameter, pore length, pore volume and part of open area, pore volume fraction, etc.

The volume porosity of fabric P_V_ can be theoretically calculated based on the filling volume of fabric F_V_ as follows [18]:

(1) $$P_{V}=100\;\%-{\;F}_{V}$$

The filling of the fabric volume is calculated as a percentage of the thread volume V_t_, with respect to the fabric volume V_f_ according to the equation [18]:

(2) $$F_{V}=\frac{V_{t}}{V_{f}}\cdot 100=\frac{m_{t}\rho_{f}}{m_{f}\rho_{t}}\cdot 100\;\%$$

where:

V_t_, V_f_—thread (t) and fabric (f) volume [cm^3^]
m_t_, m_f_— thread (t) and fabric (f) mass [g]
ρ_t_, ρ_f_ — thread (t) and fabric (f) specific gravity [g/cm^3^]

The mass of the woven fabric is actually the mass of the threads used (m_t_ = m_f_) so the previous equation can be simplified [18]:

(3) $$F_{V}=\frac{\rho_{f}}{\rho_{t}}\cdot 100\%$$

In general, all spaces filled with air can be considered as pores in the fabric. However, the porosity thus determined is usually not very appropriate in terms of investigating the relationship between the permeability of the fabric and its structure [19]. It just indicates how much air the fabric contains and says nothing about its location-the shape of the pores, their size and distribution. However, these characteristics are very important in terms of fabric permeability. Therefore, many models try to describe the geometric structure of the pores in the fabric, some of which simplify the three-dimensional structure of the fabric into a two-dimensional one. In the woven fabric, a distinction should be made between the porosity of the yarn (porosity between the threads of the yarn) and that between the fibers within the yarn (porosity within the yarn). There is an assumption that if the pores between the yarns in the woven fabric are large enough and the air has enough space for free passage, it will flow mostly that way. Therefore, in terms of air permeability assessment, porosity within the yarn is usually neglected.

**Figure 2:**
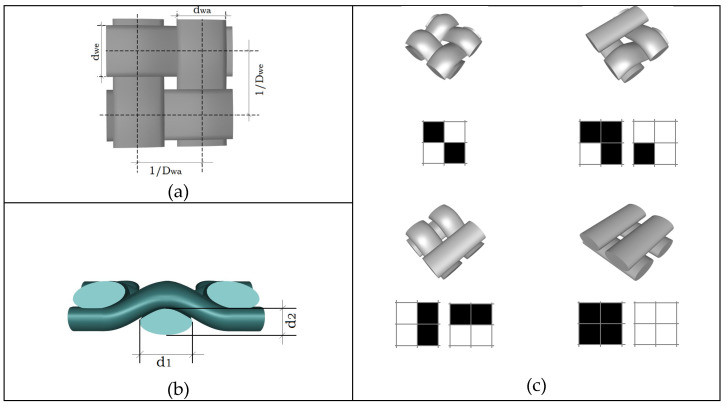
Individual woven fabric segments (**a**) yarn projection geometry; (**b**) cross section of the fabric; (**c**) three-dimensional models Adapted from [19].

The value of the yarn diameter *d* in meters is used in a number of models describing the porosity of the fabric. The yarn cross-section is flattened and distorted in the fabrics as a result of the normal forces between the yarn systems that occur during the normal weaving process. The initial, approximately circular, cross-section of the yarn is thus deformed. There are several models to describe the deformed cross-section of the yarn, for example, ellipse or lens shape. Moreover, the yarn cross-sectional shape has been shown to be variable along the length of the yarn. The type of weaving, the material and fineness of the yarn, but also the type and settings of the loom generally influences the spatial geometry of the fabric. Additionally, yarn hairiness has also been shown to affect air permeability. Experimental estimation of fabric cross-sectional parameters is relatively complex and time-consuming, but allows the measurement of deformed cross-sectional yarn in both directions (horizontal diameter d_1_ and vertical diameter d_2_—see Figure 2b.

Measuring the characteristics of the fabric plane is much easier because it allows for measuring only the horizontal diameter d_1_ [19]. Then, the vertical diameter d_2_ can be theoretically estimated under the assumption that the cross-sectional shape of the yarn in the fabric is known. Air permeability is not constant across the width of the fabric and depends on the distance from the edge of the fabric. Therefore, the diameter d_1_ is important for the accurate calculation of horizontal porosity, and both diameters-d_1_ and d_2_-are important for the calculation of vertical porosity.

## 4. Materials and Methods

### 4.1. Materials

**Figure 3:**
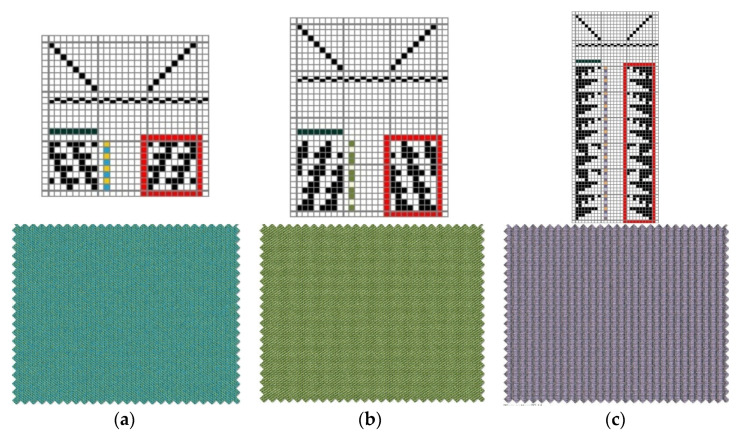
Peg plans and fabric simulation of samples (**a**) 2-weft; (**b**) 3-weft and (**c**) 4-weft.

For the purpose of the research, eight samples of fabrics were made on the laboratory weaving machine DW598 (Fanyuan Instrument-FYI)—one 2-weft (a), four 3-weft (b) and three 4-weft (c) woven fabrics. The peg plan of each samples is shown on Figure 3.

The composition of the warp was 95/5% Meta Aramid Conex NEO/Para Aramid Twaron raw yarn (hereinafter AR) with a fineness of 16.7 × 2 tex and the weft composition was 45/55% Cotton Long Staple Combed/Modacrylic Sevel FRSA/L (hereinafter MAC), fineness 25 tex. The description of the samples is shown in Table 1.

### 4.2. Methods

#### 4.2.1. Woven Fabric Porosity

**Figure 4:**
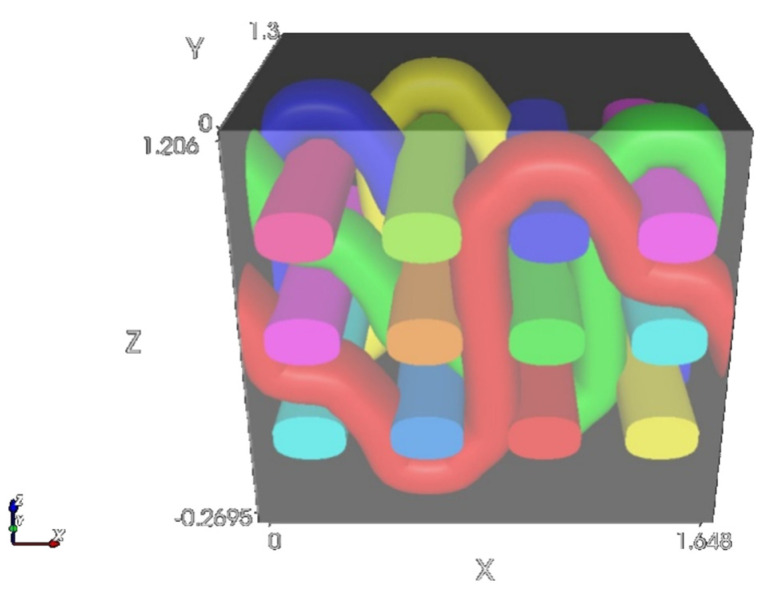
Weave unit simulation in Texgen.

The estimation of woven-fabric volume porosity was performed in the Texgen program. (Figure 4). The simulation of the weave unit of each woven sample was performed by defining measured parameters such as warp and weft thickness, average fiber density, warp and weft density and fabric thickness. The weave unit describes a square-shaped domain whose sides are in contact with the most protruding parts of the fabric on all six sides. 

From the simulation of the weave unit prepared in this way, Domain Volume Fraction (V_d_) and Yarn Fiber Volume Fraction (V_ff_) were calculated, and based on this data, the domain volume and air volume (V_a_) were calculated according to the Equations (4) and (5):

(4) $$V_{\mathrm{tex}}=V_{d}\cdot V_{\mathrm{ff}}$$

where V_tex_ stands for textile volume

(5) $$V_{a}=V_{d}-V_{\mathrm{tex}}$$

#### 4.2.2. The Resistance of Fabrics to Radiant Heat

The radiation heat transfer was tested according to EN ISO 6942 2003 with a heat flux of *Q*_0_ = 20 kW/m^2^. The test was performed on TE-08 Radiant Heat Exposure Tester. In the test procedure according to method B, the sample is exposed to a certain level of thermal radiation and time for temperature rise in calorimeters of 285.15 K (12 °C) and 297.15 K (24 °C). The obtained data were recorded and expressed as an index of heat transfer by radiation. The heat flux density across the sample is calculated according to the Equation (6) [20]:

(6) $$Q_{c}=\frac{M_{bp}\cdot Cp\cdot 12}{A\cdot \left(t_{24}-t_{12}\right)}=\frac{66,131}{\left(t_{24}-t_{12}\right)}$$

where *M_bp_* is the mass of copper plate and equals 0.036 kg, *C_p_* is the specific heat of copper which is 0.385 kJ/kg K, *A* is area of the copper plate and equals 0.002515 m^2^ and *t*_12_, *t*_24_ are the time required for a temperature rise of 285.15 K (12 °C) and 297.15 K (24 °C), respectively.

The heat-transfer factor expresses the heat flow passing during 1 h through 1 m^2^ of fabric with actual thickness, and temperature difference of two media 1 K. High *TFQ*_0_ indicates a good heat transfer (low thermal stress, i.e., good low-energy comfort at normal working conditions), while low heat transfer factor indicates a good insulation. The factor of heat transfer was calculated according to Equation (4) [20]:

(7) $$TFQ_{0}=\frac{Q_{C}}{Q_{0}}$$

where *Q_C_* is leaked heat flux density through the sample to calorimeter in kW/m^2^ and *Q*_0_ is default heat density (radiation source per calorimeter) in kW/m^2^.

#### 4.2.3. Water-Vapor Resistance

The water-vapor resistance of fabric samples was tested according to the method ISO 11,092-Textiles-Physiological effects Measurement of thermal and water-vapor resistance under steady-state conditions (sweating guarded-hotplate test). The samples were positioned on an electrically heated plate above the membrane. Distilled water was fed to the surface of the porous plate from a dosing device [21]. A piece of smooth water vapor permeability, liquid water impermeable cellophane membrane was fitted over the plate. The square porous plate was heated to a constant temperature that approximates body skin temperature. Water-vapor resistance, Ret (m^2^K/W) was calculated as a difference of a water-vapor pressure between the two faces of material divided by the resultant evaporative heat flux per unit area.

## 5. Results

This chapter presents the test results on eight designed woven fabric samples.

### 5.1. Sample Characteristics

Table 2 shows the characteristics of the woven samples and their empirical values.

### 5.2. Samples Porosity

Using the procedure described in the methodology, the volume porosity of the fabric samples was estimated, and the results are presented graphically.

**Figure 5:**
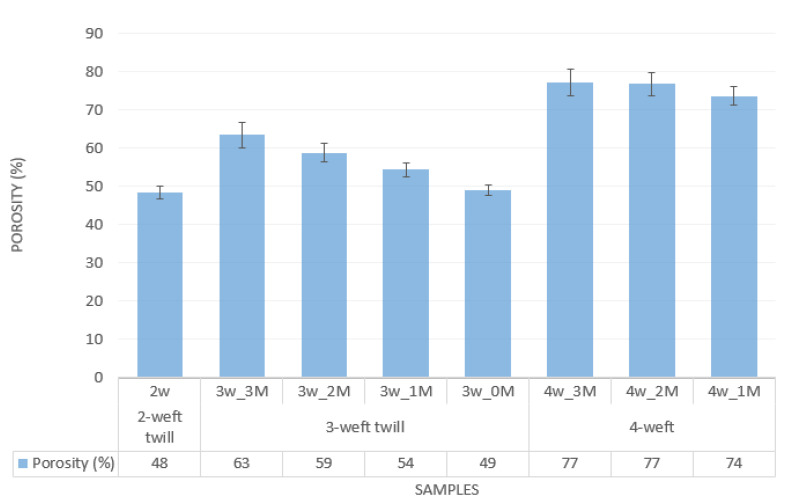
Volume porosity of the samples.

Figure 5 shows how porosity increases with the number of weft systems in the fabric. The 4-weft fabrics have the highest volume porosity values thanks to a specific structure in which a large number of flotation is present at the level of each layer. The result is a relatively loose structure that contains a large number of air pockets. The 2-weft an 3-weft fabrics are both woven in twills, which results in minor difference in volume porosity of comparable samples 2w and 3w_0M. Thus, by increasing the number of weft systems, it is possible to keep an equal proportion of pores, which enables development a woven fabric with higher thermal protective properties without significantly impairing the breathability properties. Additionally, a higher proportion of modacrylic/cotton yarn contributes to the increase in volume porosity.

### 5.3. Water Vapor Resistance

**Figure 6:**
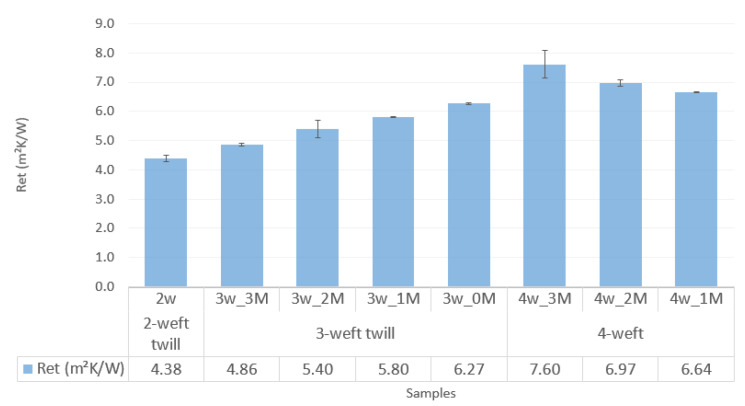
Water vapor resistance of the samples.

The graph in Figure 6 shows the results of water-vapor resistance of the samples.

Figure 6 shows the results of water vapor resistance for the 2-weft, 3-weft and 4-weft samples. The resistance to the passage of water vapor is highest in fabrics with the highest number of weft systems, i.e., in 4-weft fabrics. As the number of weft systems in fabrics decreases, the resistance to water-vapor passage through the fabric also decreases. Furthermore, with the increase in the number of aramid fibers, water vapor resistance increases with 3-weft fabrics, while the opposite is true for 4-weft fabrics due to the specific structure of the samples.

### 5.4. The Resistance of Fabrics to Radiant Heat

Table 3 shows the test results of fabric samples resistance to radiant heat.

Table 3 shows the results of 2-weft, 3-weft and 4-weft fabric resistance to radiant heat. In general, the samples with a higher number of weft systems, as well as those with a higher proportion of MAC yarn compared to aramid, which had the highest resistance to thermal radiation. Sample 4w_3M shows the highest resistance to radiant heat, the average heat flux is 6.34 kW/m^2^, and the heat transfer factor is 0.32 kW/m^2^. It took 13.73 s for the calorimeter temperature to rise from 12 °C, while 22.37 s was needed for the calorimeter temperature to rise from 24 °C. Samples 2w and 3w_0M have the lowest resistance to thermal radiation. It is obvious that the layered structure of woven fabrics has the best effect in terms of heat protection with only one aramid layer while for all others MAC is preferred.

## 6. Discussion

In this section, the obtained results are interpreted in order to identify multiple-weft system woven-fabric parameters that gives optimal comfort and thermal protection. For this purpose, the influence of structural parameters of woven fabric on the resistance to radiant heat and the influence of woven fabric mass and thickness on water-vapor resistance were analyzed.

### 6.1. Influence of Structural Parameters of Woven Fabric on Resistance of Fabrics to Radiant Heat (Multiple Linear Regression)

In order to determine the influence of the parameters of mass per area unit, weft density, fabric thickness and porosity on TFQ, statistical data processing was performed.

**Figure 7:**
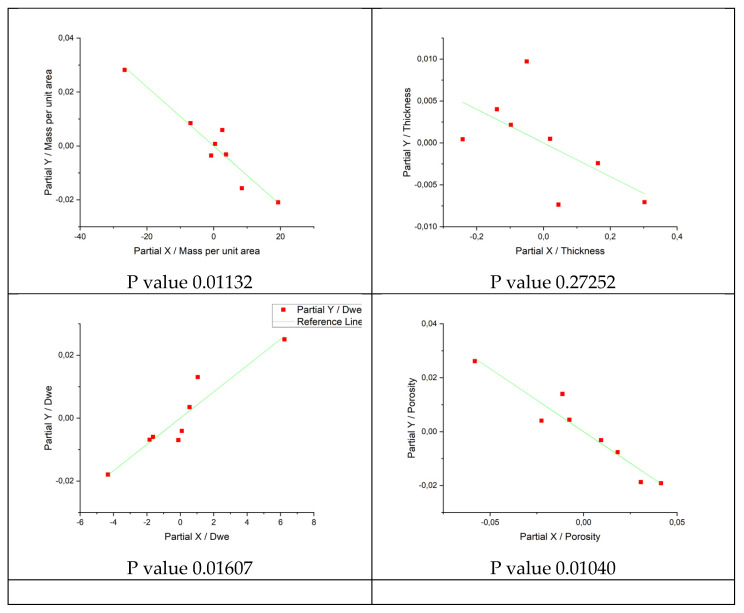
Statistics—multiple linear regression.

From the multiple regression output (Figure 7 and Table 3), ANOVA/Prob > F, it could be seen that at confidence of 0.05, this regression is statistically significant (because the *p*-value is less than 0.05). At the same confidence, all independent variables except thickness are statistically significant and it can be concluded that mass per area unit, density and porosity have a significant individual influence on TFQ, i.e., by knowing these parameters, it is possible to predict TFQ with high reliability.

From the summary table of descriptive statistics (Table 4), according to the coefficient of determination of R-Square and Adjusted R-Square, it can be seen that more than 99% of the total variation in TFQ is explained by this regression.

**Figure 8:**
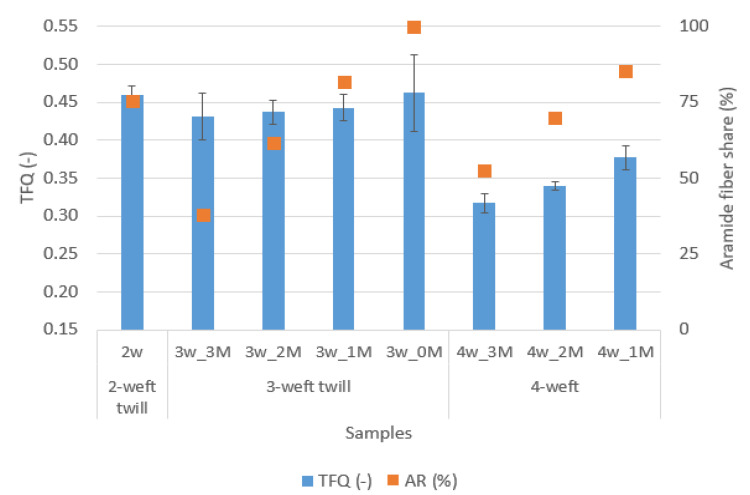
Correlation between TFQ and aramid fiber content in fabric.

**Figure 9:**
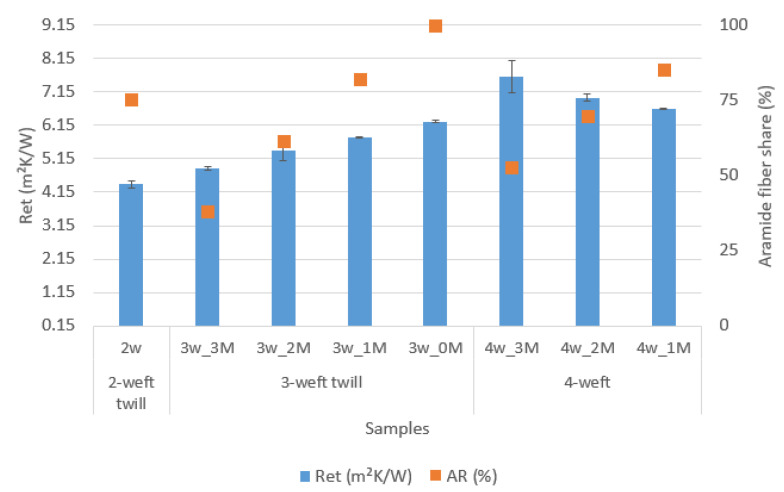
Correlation between Ret and aramid fiber content in fabric.

With the inclusion of the independent variable “AR fiber content in fabric” the *p*-values of regressors goes above 0.05 and the reason for that may be too many regressors. Additionally, there could be nonlinearity in correlations. However, two additional analyzes were performed to determine the influence of aramid fiber content in fabric on TFQ and Ret (graphs in Figure 8 and Figure 9).

The correlation between TFQ and aramid fiber content in fabric (Figure 8) is obvious, while the influence of raw material composition on Ret is questionable. 

In the graph in Figure 9 the different behavior of 3-weft and 4-weft samples can be seen, which leads to conclusion that Ret is influenced by another parameter.

For a more detailed insight into the impact of aramid fiber share on TFQ property, a linear regression was performed by two categories (3 weft and 4 weft samples).

**Figure 10:**
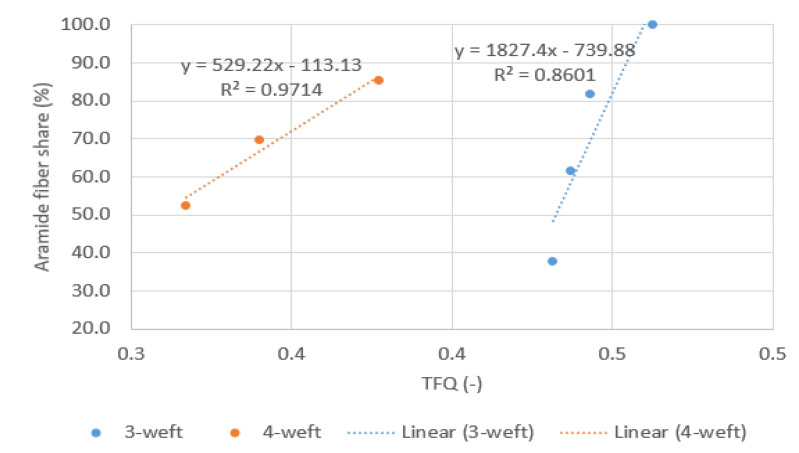
Correlation of aramid fiber share and TFQ with R^2^.

The Figure 10 shows a very high linear correlation of aramid fiber share and TFQ with R^2^ = 0.86 for 3-weft and R^2^ = 0.97 4-weft woven fabrics.

### 6.2. Influence of Woven Fabric Mass and Thickness on Water-Vapor Resistance

**Figure 11:**
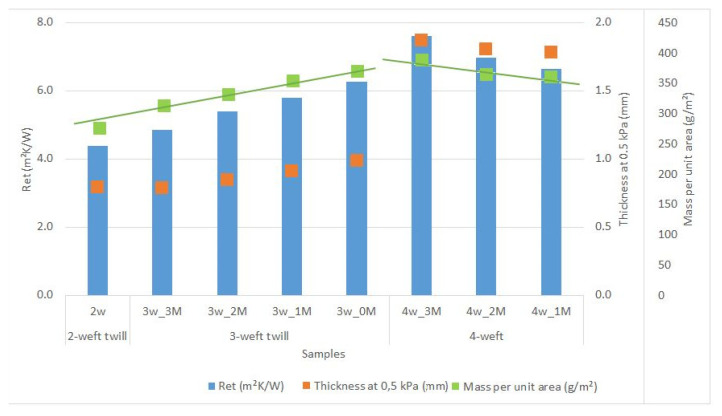
Influence of samples mass and thickness on water-vapor resistance.

The correlation between the mass and thickness of fabric samples on their resistance to water-vapor permeability is shown graphically in Figure 11.

Figure 11 shows three different categories of samples: 2-weft, 3-weft and 4-weft fabrics. The influence of the parameters thickness and mass per unit on their resistance to water vapor permeability was observed. From the results of the tested samples, it is not possible to determine exactly whether, and to what extent, the values of water-vapor resistance are the result of changes in porosity or raw material composition. The different behavior of 3-weft and 2-weft fabrics is also visible when it comes to the influence of volume porosity on water-vapor resistance. In 3-weft fabrics, by increasing the proportion of volume pores and aramid fibers in the weft, the resistance to water vapor decreases, resulting in greater breathability. With 4-weft fabrics, the situation is reversed. However, the graph in Figure 11 shows a clear correlation between the thickness and mass per unit area of fabrics on water vapor resistance, i.e., the greater the thickness and mass of the sample, the greater the water vapor resistance. Sample 4w_3M has the highest thickness and mass per unit area, which resulted in the highest water vapor resistance.

### 6.3. Correlation Table

**Figure 12:**
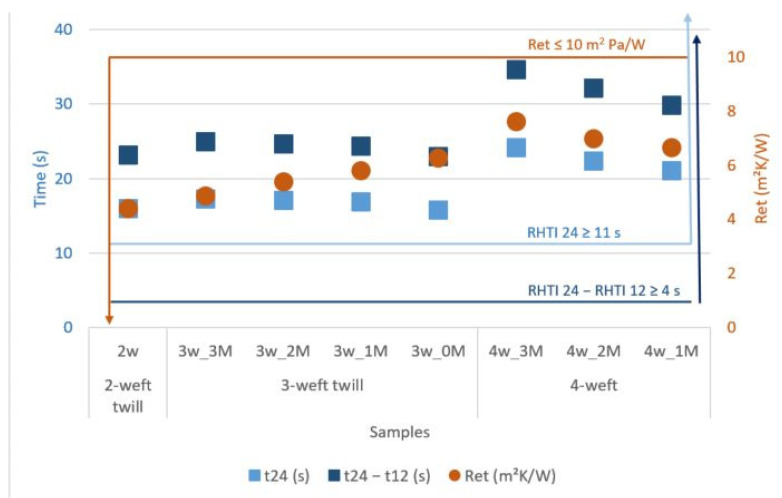
Comparison of the developed samples with the requirements of the standard.

In Figure 12, it can be seen how the individual structure parameters are linearly related to the dependent variables TFQ and Ret. 

Table 5 shows that the number of wefts system, mass per unit area, weft density, thickness and porosity are highly linearly correlated with the values of Ret and TFQ. With the increase in weft systems, mass per unit area, weft density, thickness and porosity the heat transfer factor will decrease (better protection against heat radiation) and the resistance to water vapor permeability will be higher. However, it can be seen from the Table 5 that the thickness and mass of the fabric have a greater influence on the water-vapor resistance, while the TFQ is more affected by the thickness and porosity.

### 6.4. Complianse of Multi-Weft Fabrics to the Standard Requirements

According to the requirements described in standard EN 15614: 2007 Protective clothing for fire-fighters-Laboratory test methods and performance requirements for wildland clothing, the minimum requirements for heat flux density of 20 kW/m^2^ of the single layer the component assembly or multilayer clothing assembly shall have the minimum level as follows:

- RHTI 24 ≥ 11 s
- RHTI 24-RHTI 12 ≥ 4 s,

when tested in accordance with method B of EN ISO 6942: 2002.

Additionally, the mean water vapor resistance of material or material combination (in the composite) shall give a water vapor resistance

- Ret ≤ 10 m^2^ Pa/W

when tested in accordance with EN 31092.

A comparison of the results of the developed samples with the requirements of the standard is shown graphically in Figure 12.

Figure 12 shows 2-weft, 3-weft and 4-weft fabrics and the requirements they must meet. The graph shows how all designed and produced samples meet the criteria from the standard. The 4-weft fabrics have the best thermal protection properties, while 2-weft sample have the best breathability. Taking into account both criteria, 3-weft fabric with the highest proportion of modacrylic/cotton fibers has optimal thermal protection and breathability.

## 7. Conclusions

The aim of this study was to determine the influence of structural parameters of multi-weft woven fabrics on two mutually contradictory properties—breathability and thermal protection.

Heat transmission factor is most influenced by the thread density (weft), which is structurally positioned in several layers (one above the other). By increasing the number of weft systems (and thus the thickness and mass of the fabric), the protection of fabric against thermal radiation will also increase significantly.

Depending on the porosity of the structure, the multilayered material more or less retains the property of breathability, and thus comfort. At the same time, the number of weft systems positively affects the thermal insulation properties of the fabric due to multi layering.

The ratio of aramid and modacrylic/cotton flame resistant fiber blends in fabric layers is very highly correlated with the heat transmission factor. Fabrics with a higher content of modacrylic/cotton FR blends provide better protection against heat radiation. However, in order to protect against contact flames, it is still necessary to put an aramid fiber in the outer layer of the protective material. Aramid is inherently non-combustible, and in this place protects the inner modacrylic/cotton layers from direct fire.

There is a clear correlation between the woven fabrics structural parameters (thickness and mass per unit area) and the water-vapor resistance (Ret), i.e., the greater the thickness and mass of the sample, the greater the water vapor resistance.

By manipulating weft yarns of different fiber compositions, it is possible to optimize the thermal protection and breathability properties. From the results, it can be concluded that fabrics with more layers have better thermal protection properties. At the same time the fabrics with fewer weft layers have better breathability where the porosity of the structure, which is achieved by the construction of the fabric, also plays a key role. By optimizing the number of weft systems and combining the raw material composition in such a way as to incorporate as much modacrylic/cotton FR fibers as possible into the multilayer fabric, without compromising protection against direct contact with fire, it is possible to develop a woven fabric with optimal thermal protection and comfort properties.

## Figures and Tables

**Table 1:** Description of designed and woven fabric samples.

Designation	Weave	Weft
2w	2-weft twill	1AR/1MAC
3w_3M	3-weft twill	3 MAC
3w_2M	3-weft twill	1AR/2MAC
3w_1M	3-weft twill	2AR/1MAC
3w_0M	3-weft twill	3 AR
4w_3M	4-weft	1AR/3MAC
4w_2M	4-weft	2AR/2MAC
4w_1M	4-weft	3AR/1MAC

**Table 2:** Empirical sample parameter.

Designation	Weave	Composition Aramid Fiber/Modacryl Cotton Mix Fiber (%)	Weft	Mass Per Unit Area (g/m^2^)	Density wa/we (threads/cm)	Thickness at 0.5 kPa (mm)
2w	2-weft twill	76/24	1AR + 1MAC	272	31/50	0.79
3w_3M	3-weft twill	38/62	3 MAC	308	31/71	0.79
3w_2M	3-weft twill	62/38	1AR + 2MAC	327	31/71	0.85
3w_1M	3-weft twill	82/18	2AR + 1MAC	350	30/72	0.91
3w_0M	3-weft twill	100/0	3 AR	365	30/69	0.98
4w_3M	4-weft	53/47	1AR + 3MAC	384	32/83	1.87
4w_2M	4-weft	70/30	2AR + 2MAC	360	31/81	1.80
4w_1M	4-weft	85/15	3AR + 1MAC	356	31/86	1.78

**Table 3:** Fabric resistance to radiant heat.

Weave	Designation	t_12_ (s)	t_24_ (s)	t_24_ − t_12_ (s)	Qc (kW/m^2^)	TFQ (-)
2-weft twill	2w	8.80	16.00	7.20	9.18	0.46
3-weft twill	3w_3M	9.60	17.27	7.67	8.63	0.43
	3w_2M	9.50	17.07	7.57	8.74	0.44
	3w_1M	9.40	16.87	7.47	8.86	0.44
	3w_0M	8.65	15.80	7.15	9.25	0.46
4-weft	4w_3M	13.73	24.17	10.43	6.34	0.32
	4w_2M	12.63	22.37	9.73	6.79	0.34
	4w_1M	12.27	21.03	8.77	7.54	0.38

**Table 4:** Summary table ANOVA.

Statistical Parameter	Value
R-Square	0.99264
Adj. R-Square	0.98282
Model *p*-value	0.00157

**Table 5:** Correlation table.

Property	TFQ (-)	Ret (m^2^K/W)
Number of weft systems, -	−0.90	0.90
Mass per unit area, g/m^2^	−0.74	0.92
Dwe, thread/cm	−0.79	0.85
Thickness, mm	−0.95	0.88
Porosity, %	−0.90	0.76

## Data Availability

Not applicable.
